# Text4Hope: Receiving Daily Supportive Text Messages for 3 Months During the COVID-19 Pandemic Reduces Stress, Anxiety, and Depression

**DOI:** 10.1017/dmp.2021.27

**Published:** 2021-02-08

**Authors:** Vincent I.O. Agyapong, Marianne Hrabok, Reham Shalaby, Wesley Vuong, Jasmine M. Noble, April Gusnowski, Kelly Mrklas, Daniel Li, Liana Urichuck, Mark Snaterse, Shireen Surood, Bo Cao, Xin-Min Li, Russell Greiner, Andrew J. Greenshaw

**Affiliations:** 1 Department of Psychiatry, Faculty of Medicine and Dentistry, University of Alberta, Edmonton, Canada; 2 Addiction and Mental Health, Alberta Health Services, Edmonton, Canada; 3 Cumming School of Medicine, University of Calgary, Calgary, Canada; 4 Institute of Health Economics, Edmonton, Alberta, Canada; 5 Strategic Clinical Networks™, Provincial Clinical Excellence, Alberta Health Services, Calgary, Canada; 6 Department of Community Health Sciences, Cumming School of Medicine, University of Calgary, Calgary, Canada; 7 Department of Computing Science, Faculty of Science, University of Alberta, Edmonton, Canada; 8 Asia-Pacific Economic Cooperation (APEC) Digital Hub for Mental Health, Canada

**Keywords:** community mental health services, emergency preparedness, health policy, mental disorders, psychological resilience

## Abstract

**Background:**

This study reports on the changes in stress, anxiety, and depressive symptoms of subscribers after 3 months using Text4Hope, a supportive text messaging program designed to provide support during the pandemic.

**Methods:**

Standardized self-report measures were used to evaluate perceived stress (measured with the Perceived Stress Scale-10 [PSS-10]), anxiety (measured with the General Anxiety Disorder Scale 7 [GAD-7]), and depressive symptoms (measured with the Patient Health Questionnaire [PHQ-9]), at baseline and 3rd month (*n* = 373).

**Results:**

After 3 months of using Text4Hope, subscribers’ self-reports revealed significant (*p*< 0.001) mean score reductions compared with baseline on: the GAD-7 by 22.7%, PHQ-9 by 10.3%, and PSS-10 scores by 5.7%. Reductions in inferred prevalence rates for moderate to high symptoms were also observed, with anxiety demonstrating the largest reduction (15.7%).

**Conclusions:**

Observed Text4Hope-related reductions in psychological distress during COVID-19 indicate that Text4Hope is an effective, convenient, and accessible means of implementing a population-level psychological intervention.

Coronavirus disease 2019 (COVID-19), an acute respiratory disease, was first reported in December 2019 in Wuhan, China. The virus has spread internationally and, through late 2020, continues to have unprecedented impact on our health and ways of life. The closing of schools and businesses, extremely high unemployment rates, and the effect of quarantine are additional stressors due to public health measures in place to limit spread of COVID-19. There are significant psychological effects of the pandemic^1,2^ that affect the general population and may be more pronounced in certain groups (e.g. female, socially stressed, frontline worker, pre-existing psychological disorder; see Lai et al^3^). Provision of support for these challenges are complicated by the high number of people requiring support and the need to maintain physical distancing.

Mobile health technology offers a unique and innovative solution in this context. Specifically, this tool offers a convenient, cost-effective, and accessible means for implementing population-level interventions. Smartphone ownership is prevalent in Canada, text-messaging is free to end-users, does not require technical skill for use, and does not require expensive data plans. Text messages are also cost-effective to providers, costing cents per message to deliver.^4^ Use of supportive text messages has shown positive outcomes in randomized controlled trials, including reduction of depressive symptoms,^5^ increased abstinence duration in alcohol use disorder,^5^ and high user satisfaction evinced by previous research.^6^

This study describes effects of implementation of the Text4Hope program,^7^ a low-cost, evidence-based, supportive text messaging service free to all Canadians who wish to subscribe during the early phase of the COVID-19 pandemic. Baseline data collected at the start of messaging indicated that the majority of subscribers endorsed elevated levels of stress and depressive and anxiety symptoms.^8^ The primary aim of the study was to assess whether the Text4Hope program would reduce stress, anxiety, and depressive symptoms at the third-month follow-up. A randomized controlled trial of daily supportive text messaging resulted in close to a 25% additional improvement in Becks Depression Inventory scale-measured mood at the third-month follow-up assessment in the intervention group compared with the control group.^5^ Based on this, the study hypothesis was that the Text4Hope intervention would result in ≥25% reduction in mean scores and prevalence rates in all 3 factors: the Perceived Stress Scale-10 (PSS-10), General Anxiety Disorder Scale 7 (GAD-7), and Patient Health Questionnaire (PHQ-9) scales at 3rd month versus baseline. This study is part of a larger project,^9^ with additional results forthcoming. A literature search on major scientific data bases, including; MEDLINE, Scopus, Embase, Web of Science, Google Scholar, Chemical Abstracts, and PsychINFO, suggests that, this is the first study to report 3rd-month outcomes for a supportive text message program which seeks to address stress, anxiety, and depression at the population level during a pandemic.

## Methods

Complete methods details, including sample size estimations and citations for standardized scales are provided in the published protocol.^9,10^ In summary, this cross-sectional study was approved by the Research and Ethics Board of the University of Alberta (Pro00086163). Participation was voluntary; individuals self-subscribed to receive daily supportive text messages for 3rd month by texting the word “COVID19HOPE” once to a specified number. This program was launched through an announcement by Alberta’s Chief Medical Officer of Health on behalf of Alberta Health Services and the Government of Alberta on March 23, 2020. The announcement was widely broadcast across many electronic and print media networks in Alberta to inform Albertans about the program.^11^ Albertans were further made aware of the program by means of websites dedicated to the service (https://www.albertahealthservices.ca/topics/Page17019.aspx and https://mentalhealthfoundation.ca/text4hope/), electronic media, social media feeds, posters at addiction and mental health clinics, emergency departments and wards, and through word of mouth. The messages were aligned with a cognitive behavioral framework, with content written by mental health professionals and co-authors (V.I.O.A. who is a psychiatrist and M.H. who is a Clinical Psychologist). Most of the messages were adopted or modified from messages used in 2 randomized controlled trials in Alberta,^12,13^ and also the Text4Mood program, which reported positive effects on the mental wellbeing of Albertans and achieved high satisfaction rates.^4^ The messages were delivered to subscriber cell phones daily at 9 am Mountain Time. Each subscriber received the Text4Hope daily messages for 3 months, with the option to subscribe for an extended 6-mo Text4Mood program on completion of the Text4Hope program. Examples of the Text4Hope text messages include:

- Put yourself on a media diet. It is important to stay informed, but only check the news and social media intermittently, rather than continuously.
- Take a moment to notice how you feel right now. Do not judge your emotions or try to change them. Just observe them and see your stress levels reduce.
- Make yourself a “coping kit.” Include healthy things that help you feel better like music, inspirational messages, or a friend’s number.

Subscribers were sent a link to the online survey by means of the first text messages they received the same day after subscribing to the program and invited to complete a baseline survey that assessed their mental wellbeing using validated scales for stress, anxiety, and depression. Follow-up surveys were sent by means of text messages to all subscribers 6th week and 3rd month after they started receiving daily supportive text messages. Consent was implied if subscribers accessed, completed, and submitted their responses to the online survey. No personally identifiable information apart from subscriber phone numbers was collected, and phone numbers were only used to link the baseline data with the 6th week and 3rd month data for individual subscribers to facilitate measurement of change in the psychometric scales. Data were collected using the Survey Select tool, and extracted data were stored without the identifying phone numbers on a password protected computer. Primary outcome measures at 3rd month were the mean difference in scores on the PSS-10,^14^ GAD-7 scale,^15^ and the PHQ-9, respctively.^16^

With a prediction that daily supportive text messages would result in a 25% reduction in mean PSS-10, GAD-7, and PHQ-9 scores at 3rd month from baseline, a population variance of 5.0 for each scale mean score, a 1-sided significance level α = 0.05, and an acceptable difference between sample mean and population mean score for each scale of zero (μ-μ0 = 0), the estimate was that a sample size of 686 would be sufficient to detect mean differences between the baseline and 3rd month PSS-10, GAD-7, and PHQ-9 scores with a power of 80% (β = 0.2). Data analysis was undertaken using IBM Statistical Package for Social Sciences (SPSS) Statistics for Windows, version 26. Paired t-tests were used to assess differences between the mean PSS-10, GAD-7, and PHQ-9 scale scores at baseline and 3rd month for subscribers who completed the instruments at both time points. In addition, Chi-Square test was used to compare prevalence rates for perceived stress, likely GAD, and likely MDD at baseline and 3rd month. Moderate or high perceived stress, likely GAD, and likely MDD were assessed using cut-off scores of PSS-10 ≥ 14, GAD-7 ≥ 10, and PHQ-9 ≥ 10, respectively.^9^ There was no imputation for missing data and totals reported represent total responses recorded for each variable.

**Figure 1.**
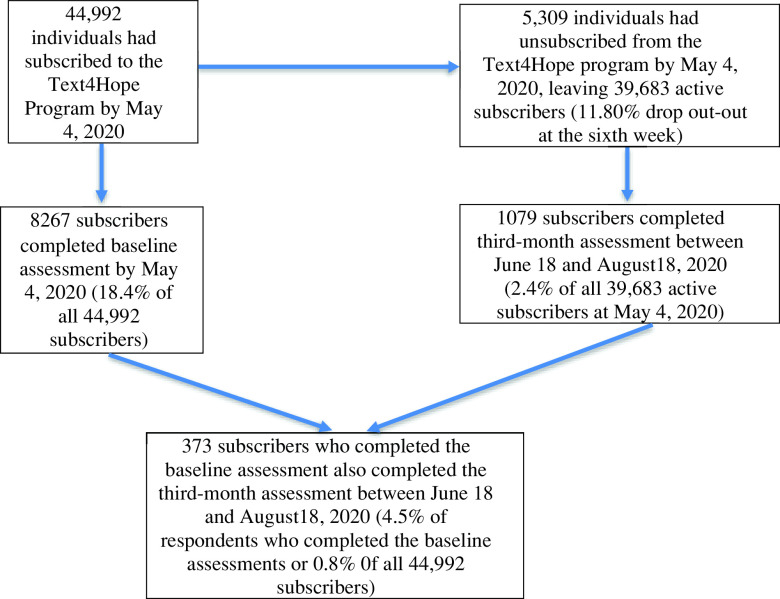
Subscriber flowchart.

Figure 1 is the study flow chart for individuals who subscribed to Text4Hope between March 24, 2020, and May 4, 2020.

## Results

The majority of the 1079 subscribers who responded to the 3rd month surveys were female (*n* = 953; 89.1%), 26 to 60 years of age (*n* = 824; 78.3%), Caucasian (*n* = 883; 83.4%), homeowners (*n* = 638; 71.8%), had postsecondary education (*n* = 825; 92.1%), and were employed (*n* = 631; 70.3%).

Table 1 presents changes in primary outcome measures after 3rd month compared with baseline. The data indicate that mean scores on each of the PSS-10, PHQ-9, and GAD-7 scales were significantly lower at 3rd month compared with mean scores at baseline, suggesting improvement in stress, depression, and anxiety symptoms. The largest reduction in mean scores at 3rd month compared with baseline scores was on GAD-7 (-22.7%) followed by PHQ-9 (-10.3%) and then PSS-10 (-5.7%). The data of Table 1 indicate statistically significant reductions in the inferred prevalence rates for moderate/high stress, depressive symptoms, and anxiety symptoms comparing baseline and 3rd month assessments. Anxiety was associated with the largest inferred prevalence rate reduction (15.7%).

**Table 1.** Comparison of the baseline and third-month mean scores on the PSS-10, PHQ-9, and GAD-7 and the prevalence rates of moderate or high stress, likely MDD, and likely GAD*^+^

Measure	*N*	Baseline Scores	Third-month Scores	Mean Difference (SD)	95% Confidence Interval	df	t-value	*P*−value	Effect size (Cohen’s d)
		Mean (SD)	Mean (SD)						
PSS-10	335	20.21(7.23)	19.07(7.74)	1.149 (6.317) −5.7% from baseline	0.47-1.83	334	3.33	<0.001	
PHQ-9	302	9.32(6.23)	8.36 (6.62)	0.96 (5.28) −10.3% from baseline	0.36-1.56	301	3.16	0.002	
GAD-7	292	9.07(6.02)	7.01(5.84)	2.06 (4.92) −22.7% from baseline	1.49-2.62	292	7.16	<0.001	
* Not all subscribers completed all 3 scales; therefore, the *N* for each scale is less than the Total *N*.									

Variables	Baseline Moderate or High Stress prevalence	Third-Month Moderate or High Stress prevalence	Baseline Likely MDD prevalence	Third-Month Likely MDD prevalence	Baseline Likely GAD prevalence	Third-Month Likely GAD prevalence
***N*** **(%)**	272 (81.2%)	249 (74.3%)	132 (43.7%)	102 (33.8%)	119 (40.6%)	73 (24.9%)
% change in prevalence rates from baseline	−6.9%		−9.9%		−15.7%	
Chi squared	73.73		55.67		65.17	
*P*-value	<0.001		<0.001		<0.001	

+ Moderate or high perceived stress, likely GAD, and likely MDD were assessed using cutoff scores of PSS-10 ≥ 14, PHQ-9 ≥ 10, and GAD-7 ≥ 10, respectively.

## Discussion

The impact of COVID-19 on health, way of life, and psychological safety and wellbeing is difficult to overstate. The threat posed by the pandemic to psychological well-being requires use of innovative techniques that can serve the high number of people requiring support while respecting the need to maintain physical distancing. Text4Hope was designed to provide mental health support on a Provincial (Canada) scale during the COVID-19 pandemic. This study examines changes in stress, depression, and anxiety symptoms after 3rd month of receiving Text4Hope messages from this low-cost, evidence-based, scalable, supportive messaging service that was delivered at no direct cost to end users. Although self-reported levels of stress, anxiety, and depressive symptoms remained high overall following the study period, both the mean scores for stress, anxiety and depression on standardized scales and the prevalence rates for clinically meaningful stress, anxiety, and depression showed statistically significant reductions (6% to over 20%; *P* < 0.01). The significant reductions in depression symptoms in particular are consistent with results reported in previous randomized controlled trials of supportive text message interventions for the treatment of major depressive disorder.^5,12^ Significant reductions in anxiety with supportive text messaging were also reported in other randomized controlled clinical trials.^17^ This study did not, however, achieve the >25% reduction in stress, depression, or anxiety mean scores at the third month as stated in the study hypothesis. It is possible that levels of stress, anxiety, and depression remain high due to overall media coverage of surges in COVID infections. It is also possible that levels of stress, anxiety, and depression will improve further if subscribers continue to receive supportive text messages for a few more months. This is why subscribers completing the 3rd month intervention are offered information on how to subscribe for 6 additional months of supportive text messages through the Text4Mood program. As of December 20, 2020, over 5000 individuals who had completed the 3rd month Text4Hope program had enrolled on the 6th month Text4Mood program to receive additional support. Evaluation of this extended text support is underway to assess possible incremental benefits to subscribers who opt to join this program.

Limitations of the present study include the very low response rate (4.5% of subscribers who completed baseline assessments and 0.8% of all subscribers), the relatively small sample size and missing data, which could lead to sampling error. It is possible that subscribers who did not participate in the surveys and those who provided incomplete responses might have different 3rd month outcomes compared with those who fully completed both surveys. Furthermore, it is possible that the demographic and baseline clinical characteristics of those who completed the assessments at both time points could be different from those of the large number of Text4Hope subscribers who completed the baseline assessments (*n* = 8267). However, a related study^8^ published by this research group, which examined the baseline demographic and clinical characteristics of the larger sample of subscribers who completed the baseline assessments (*n* = 8267), had similarities with the baseline characteristics of participants who were studied as part of this 3rd month evaluation of Text4Hope (*n* = 1079). For example, in terms of demographic characteristics, there were 87.1% versus 89.1% female gender, 77.1% versus 78.3% aged 26-60 y, 82.3% versus 83.4% Caucasians, 85.2% versus 92.1% postsecondary education and 73.4% versus 70.3% employed for all subscribers who completed baseline assessments (*n* = 8267)^8^ and subscribers who completed the third-month assessments (*n* = 1079), respectively. In terms of clinical characteristics at baseline, the mean PSS score was 20.79 (standard deviation [SD] = 6.83; *n* = 7589) vs. 20.21 (SD = 7.23; *n* = 335), mean PHQ-9 scores was 9.68 (SD = 5.87; *n* = 7082) vs. 9.32 (SD = 6.23; *n* = 302), and the mean GAD score was 9.43 (SD = 6.29; *n* = 6944) vs. 9.07(6.02; *n* = 292) for all subscribers who completed baseline assessments and subscribers who completed both the baseline and third-month assessments, respectively.^8^

Another limitation is the lack of a control group who did not receive the Text4Hope intervention. It is possible, that stress, anxiety, and depression levels would have naturally decreased over time without the intervention. This is plausible as the reported daily mean new COVID-19 infections in Alberta, calculated using an SPSS program from the officially reported daily new infections, had reduced marginally from 104 (SD = 77) during the baseline data collection time period to 78 (SD = 33) during the 3rd month data collection period.^18^ It should be noted that a natural history of improvement is unlikely, however, as the majority of Canadians recently surveyed reported that their mental health is the same or has worsened since the initial COVID-19 wave.^19^

Finally, the study used self-reported questionnaires for assessing symptomatology and, therefore, lacked comprehensive assessment to evaluate whether or not symptomatology reported met criteria for clinically significant mental health conditions. The study sample also evidenced multiple protective factors, including high levels of education and employment, and the sample was predominantly female. Therefore, it is unclear how findings from this study may generalize to other demographic groups.

## Conclusions

Limitations notwithstanding, the results from this study support the proposal that public health interventions during pandemics may benefit from mental health wellness campaigns aimed at reducing psychological impacts. This study may serve to provide evidence-based support for such policy implementation in high-, middle-, and low-income countries. The research team, therefore, plans to explore national scale-up and implementation of the Text4Hope program in multiple languages to benefit all Canadians. The team will also disseminate this program for adaptation and potential global use through the E-Text4PositiveMentalHealth platform, currently under development, and formation of partnerships with national and regional health authorities and institutions.

## References

[1] Wang C , Pan R , Wan X , et al. Immediate psychological responses and associated factors during the initial stage of the 2019 coronavirus disease (COVID-19) epidemic among the general population in China. Int J Environ Res Public Health. 2020;17(5):1729. doi: 10.3390/ijerph17051729 PMC7084952 32155789

[2] Brooks SK , Webster RK , Smith LE , et al. The psychological impact of quarantine and how to reduce it: rapid review of the evidence. Lancet. 2020;395(10227):912–920. doi: 10.1016/S0140-6736(20)30460-8 32112714 PMC7158942

[3] Lai J , Ma S , Wang Y , et al. Factors associated with mental health outcomes among health care workers exposed to coronavirus disease 2019. JAMA Netw Open. 2020;3(3):e203976. doi: 10.1001/jamanetworkopen.2020.3976 32202646 PMC7090843

[4] Agyapong VIO , Mrklas K , Juhás M , et al. Cross-sectional survey evaluating Text4Mood: mobile health program to reduce psychological treatment gap in mental healthcare in Alberta through daily supportive text messages. *BMC Psychiatry*. 2016;16(1). doi: 10.1186/s12888-016-1104-2 PMC5100254 27821096

[5] Agyapong VIO , Ahern S , McLoughlin DM , et al. Supportive text messaging for depression and comorbid alcohol use disorder: single-blind randomised trial. J Affect Disord. 2012;141(2-3):168-176. doi: 10.1016/j.jad.2012.02.040 22464008

[6] Agyapong VIO , Milnes J , McLoughlin DM , et al. Perception of patients with alcohol use disorder and comorbid depression about the usefulness of supportive text messages. Technol Health Care. 2013;21(1):31-39. doi: 10.3233/THC-120707 23358057

[7] Agyapong VIO. Coronavirus disease 2019 pandemic: health system and community response to a text message (Text4Hope) program supporting mental health in Alberta. Disaster Med Public Health Prep. 2020;14(5):e5-e6. doi: 10.1017/dmp.2020.114 PMC7198462 32317038

[8] Nwachukwu I , Nkire N , Shalaby R , et al. COVID-19 pandemic: age-related differences in measures of stress, anxiety and depression in Canada. Int J Environ Res Public Health. 2020;17(17):6366. doi: 10.3390/ijerph17176366 PMC7503671 32882922

[9] Agyapong V , Hrabok M , Vuong W , et al. Closing the psychological treatment gap during the COVID-19 pandemic with a supportive text messaging program: protocol for implementation and evaluation. JMIR Res Protoc. 2020;9(6):e19292. doi: 10.2196/19292 32501805 PMC7309448

[10] Agyapong VIO , Hrabok M , Vuong W , et al. Changes in stress, anxiety, and depression levels of subscribers to a daily supportive text message program (Text4Hope) during the COVID-19 pandemic: cross-sectional survey study. JMIR Ment Health. 2020;7(12):e24323. 10.2196/22423 PMC7752184 33296330

[11] Pearson H. Coronavirus: new texting initiative gives Albertans mental health support. Global News 2020. https://globalnews.ca/news/6722244/coronavirus-alberta-mental-health-support-texts/. Accessed December 26, 2020.

[12] Agyapong VIO , Juhás M , Omeje J , et al. Randomized controlled trial of supportive text messages for patients with depression. BMC Psychiatry. 2017;17(1):286. doi: 10.1186/s12888-017-1448-2 28768493 PMC5541655

[13] Agyapong VIO , Juhás M , Mrklas K , et al. Randomized controlled pilot trial of supportive text messages for patients with alcohol use disorder. J Subst Abuse Treat. 2018;94:74-80. doi: 10.1016/j.jsat.2018.08.014 30243421

[14] Cohen S , Kamarck T , Mermelstein R. A global measure of perceived stress. J Health Social Behav. 1983;24(4):385-396. 6668417

[15] Spitzer RL , Kroenke K , Williams JBW , et al. A brief measure for assessing generalized anxiety disorder: the GAD-7. Arch Intern Med. 2006;166(10):1092-1097. 16717171 10.1001/archinte.166.10.1092

[16] Kroenke K , Spitzer RL , Williams JB. The PHQ-9: validity of a brief depression severity measure. J Gen Intern Med. 2001;16(9):606-613. 11556941 10.1046/j.1525-1497.2001.016009606.x PMC1495268

[17] Kwan MK , Chiu CK , Gan CC , et al. Can intraoperative text messages reduce parental anxiety of children undergoing posterior spinal fusion surgery for adolescent idiopathic scoliosis? Spine (Phila Pa 1976). 2016;41(4):E225-E230. doi: 10.1097/BRS.0000000000001199 26579957

[18] Government of Alberta. COVID-19 Alberta statistics- Interactive aggregate data on COVID-19 cases in Alberta. https://www.alberta.ca/stats/covid-19-alberta-statistics.htm#data-export. Accessed December 21, 2020.

[19] Global News. 25% of Canadians say their mental health is worse than in 1st coronavirus wave: poll. https://globalnews.ca/news/7407408/canadians-mental-health-first-coronavirus-wave/. Accessed October 28, 2020.

